# Identification of stages of amelogenesis in the continuously growing mandiblular incisor of C57BL/6J male mice throughout life using molar teeth as landmarks

**DOI:** 10.3389/fphys.2023.1144712

**Published:** 2023-02-10

**Authors:** Ai Thu Bui, Lyudmila Lukashova, Kostas Verdelis, Brent Vasquez, Lasya Bhogadi, Claire M. Gabe, Henry C. Margolis, Elia Beniash

**Affiliations:** ^1^ Department of Oral and Craniofacial Sciences, University of Pittsburgh School of Dental Medicine (UPSDM), Pittsburgh, PA, United States; ^2^ Center for Craniofacial Regeneration, UPSDM, Pittsburgh, PA, United States; ^3^ Department of Endodontics, UPSDM, Pittsburgh, PA, United States; ^4^ Department of Periodontics and Preventive Dentistry, UPSDM, Pittsburgh, PA, United States

**Keywords:** amelogenesis, microdissection, aging, molar landmarks, transition, apical movement

## Abstract

Continuously growing mouse incisors are widely used to study amelogenesis, since all stages of this process (*i.e*., secretory, transition and maturation) are present in a spatially determined sequence at any given time. To study biological changes associated with enamel formation, it is important to develop reliable methods for collecting ameloblasts, the cells that regulate enamel formation, from different stages of amelogenesis. Micro-dissection, the key method for collecting distinct ameloblast populations from mouse incisors, relies on positions of molar teeth as landmarks for identifying critical stages of amelogenesis. However, the positions of mandibular incisors and their spatial relationships with molars change with age. Our goal was to identify with high precision these relationships throughout skeletal growth and in older, skeletally mature animals. Mandibles from 2, 4, 8, 12, 16, and 24-week-old, and 18-month-old C57BL/6J male mice, were collected and studied using micro-CT and histology to obtain incisal enamel mineralization profiles and to identify corresponding changes in ameloblast morphology during amelogenesis with respect to positions of molars. As reported here, we have found that throughout active skeletal growth (weeks 2–16) the apices of incisors and the onset of enamel mineralization move distally relative to molar teeth. The position of the transition stage also moves distally. To test the accuracy of the landmarks, we micro-dissected enamel epithelium from mandibular incisors of 12-week-old animals into five segments, including 1) secretory, 2) late secretory - transition - early maturation, 3) early maturation, 4) mid-maturation and 5) late maturation. Isolated segments were pooled and subjected to expression analyses of genes encoding key enamel matrix proteins (EMPs), *Amelx*, *Enam*, and *Odam*, using RT-qPCR. *Amelx* and *Enam* were strongly expressed during the secretory stage (segment 1), while their expression diminished during transition (segment 2) and ceased in maturation (segments 3, 4, and 5). In contrast, *Odam*’s expression was very low during secretion and increased dramatically throughout transition and maturation stages. These expression profiles are consistent with the consensus understanding of enamel matrix proteins expression. Overall, our results demonstrate the high accuracy of our landmarking method and emphasize the importance of selecting age-appropriate landmarks for studies of amelogenesis in mouse incisors.

## 1 Introduction

Determining the changes in enamel mineral formation as a function of ameloblast differentiation and changes in gene expression are key to our understanding of amelogenesis. Murine incisors are hypselodont teeth and grow continuously throughout their lifespan, unlike most other mammalian teeth, which complete their crown formation before eruption (Renvoisé and Michon, 2014). Accordingly, all stages of enamel formation (amelogenesis) are present in a single continuously growing murine incisor in a spatially determined sequence (Smith et al., 1996; Verdelis et al., 2016). Due to these unique features, murine incisors are widely used to study amelogenesis. The continuously growing murine incisor has served as a reliable model to study enamel formation from a variety of perspectives (Smith et al., 1996; Smith et al., 2005; Beniash et al., 2009; Bui et al., 2022). The development of genetic tools to specifically target and edit genes made it possible to answer mechanistic questions about the functions of EMPs and other ameloblast machinery and to better understand hereditary diseases of enamel (Gibson et al., 2001; Caterina et al., 2002; Shin et al., 2020; Liang et al., 2022). Murine models have also been used to study the effects of environmental factors on enamel formation (Jedeon et al., 2014; Jalali et al., 2017; Bui et al., 2022).

Ameloblasts are highly specialized cells, which perform specific functions, depending on the stage of amelogenesis. They undergo a complex histodifferentiation process, comprising several discrete and functional stages of amelogenesis–*i.e.,* presecretory, secretory, transition and maturation (Hu et al., 2007; Lacruz et al., 2012a; Lacruz et al., 2017; Krivanek et al., 2020). Therefore, reliable methods of collecting ameloblasts from different stages of amelogenesis are required when studying the biological roles of stage-specific ameloblasts in enamel formation, for example, through the comparison changes in patterns of gene and protein expression in association enamel mineral deposition.

The collection of distinct ameloblast populations using micro-dissection relies on the stable positions of molar teeth as landmarks for identifying the different stages of amelogenesis in mouse incisors. Micro-dissection was first developed in the rat model (Hiller et al., 1975; Smith and Nanci, 1989), which is more suitable for enamel organ tissue collection than mice because of the larger size of rat teeth. However, due to the widespread use of mice as an animal model for genetic modifications, they are commonly used to study genetic bases of dental tissue development and disease (Brookes et al., 2017; Shin et al., 2020; Liang et al., 2022). Likewise, in studies of complex environmental effects on dental development when multiple treatment conditions and/or large sample sizes are needed, mice are a preferable choice when compared to other rodents (Bui et al., 2022). While micro-dissection methods for rat enamel organs are well established (Smith and Nanci, 1989; Smith et al., 1996; Moffatt et al., 2008; Lacruz et al., 2012a; Lacruz et al., 2012b; Jedeon et al., 2014), they still require further refinement in mice. Only a handful of studies that utilize micro-dissection techniques have been conducted on mice (Smith et al., 2005; Smith et al., 2011; Houari et al., 2018; Bui et al., 2022). However, these were performed on animals of a single age and the resolution of the micro-dissection techniques used in these studies was fairly coarse. More importantly, the impact of mouse age on the positions of the incisors in mandibles and their spatial relationships with molar teeth has not been fully addressed, especially during the period of skeletal growth (Wei et al., 2017), a factor that could markedly affect findings, particularly when conducting longitudinal studies. The goal of this study was to develop a methodology to collect ameloblasts from different stages of enamel formation in mouse incisors with high precision and over the lifespan of the animal during the active skeletal growth phase, and in skeletally mature animals.

## 2 Materials and methods

### 2.1 Animals and sample collection

Male C57BL/6J mice (Jax^®^ #000664) were bred and raised in the Division of Laboratory Animal Resources at the University of Pittsburgh, following protocols approved by the Institutional Animal Care and Use Committees, University of Pittsburgh. All mice were offered a mixture of regular hard chow (LabDiet, 5P76-Prolab Isopro RMH 3000) and gel food (Diet Gel 76AGel, ClearH_2_O), under a 12-h dark-light cycle. 2-week-old (wo), 4-wo, 8-wo, 12-wo, 16-wo, 24-wo, and 18-month-old (mo) mice were used in this study. Each group contained three to five mice from different breeding pairs. A total of 51 mice were used in this study (Supplementary Table S1). Of those, 27 mice were collected for micro-CT analyses and histology, including two 12-wo and two 16-wo male mice that were obtained from Jackson Laboratory (from facilities in Ellsworth, Maine or Bar Harbor, Maine, United States), which were kept on a regular hard diet (LabDiet, 5P76-Prolab Isopro RMH 3000) only (Supplementary Table S1). Another 24 12-wo male mice from our colony were used for micro-dissection and RNA collection (Supplementary Table S1).

### 2.2 Micro CT analysis

Mice were euthanized with carbon dioxide, followed by decapitation. The hemimandibles were immediately cleaned of any attached soft tissues. Left hemimandibles were stored in 70% ethanol (Decon Labs 2,701) at 4°C. Left hemimandibles were scanned using a Scanco µCT 50 scanner (Scanco Medical, Brüttisellen, Switzerland) system at 6 µm voxel size, 55 KVp, 145 μA, a 0.36° rotation step (180° angular range), and a 1,500 ms exposure time per view.

Samples were orientated identically with the scan plane perpendicular to the distal root of the M2 molar to minimize the curvature of the incisor in the zone of interest (0–4 mm from the apex), using Scanco µCT software (HP, DECwindows Motif 1.6) for 3D reconstruction and viewing of images. The positions of the first, second and third molars (M1, M2, and M3, respectively) were recorded. The enamel layer was then contoured, segmented and analyzed using a threshold set at 180 mgHA/cm^3^. Mineral density distribution was recorded from the cervical end toward the peak. Enamel volume (EV) profile of each enamel block of 6 µm-thickness was recorded also from the cervical end toward the peak, using the formula: EV (µm^3^) = EA × 6 μm, where EA is the area of each scanned enamel slice. Illustrations of sagittal and longitudinal sections, as well as 3D reconstructions, were obtained *via* Data Viewer software (Skyscan, release 1.5.2.4, Kontich, Belgium).

### 2.3 Trichrome masson staining

**FIGURE 1:**
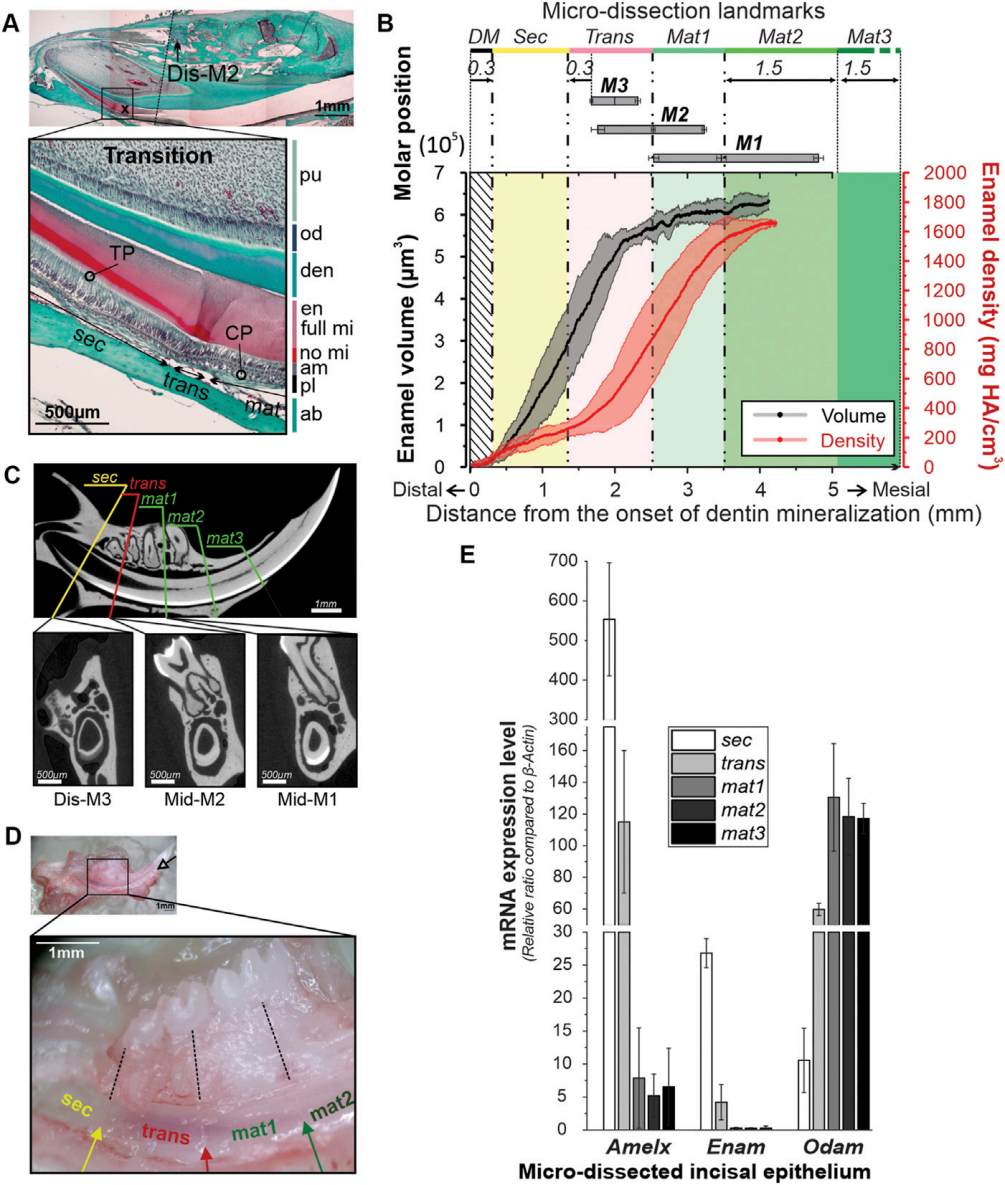
Identification of skeletal landmarks of the amelogenesis in mandibular incisors in 12-week-old mice. **(A)** Trichrome Masson staining of the mandible of a 12-wo mouse showing the correlation between transition stage ameloblasts and molar positions. ab, alveolar bone; am, ameloblasts; CP, capillaries; den, dentin; en, enamel; full mi, fully mineralized; no mi, partially mineralized; od, odontoblasts; TP, Tomes’ processes; sec, secretory ameloblasts; trans, transition ameloblasts, mat, maturation ameloblasts; **(B)** average enamel volume (EV) profile (black line) and the mineral density (MD) profile (red line) from the apex toward the incisal tip. The grey and pink bands surrounding the EV and MD profiles represent ± SDs. Positions of M1, M2 and M3 represented by bars with the means and SDs of the distal, mid- and mesial planes. For definitions of Sec, Trans and Mat1-3 strips see the text. **(C)** MicroCT analysis of the skeletal landmarks. Top image shows a midsagittal section through an incisor with five lines identifying five dissection planes in respect to the positions of molar teeth. The bottom microCT transverse sections correspond to sec/trans, trans/mat1 and mat2/mat3 dissection planes. **(D)** Mandible of a male 12-wo mouse at low, X2.5, (top) and higher, ×5, magnification, showing markings on the lingual face of the incisor. Markings separating sec, trans, mat1, and mat2 segments were made in the planes intersecting distal plane of M3, mid-M2, and mid-M1, and normal to the incisor surface. Enamel organ was separated into strips based on the markings and collected for gene expression analysis. **(E)** Bar graph showing mean expression levels of *Amelx, Enam*, and *Odam*, in the five micro-dissected enamel organ strip samples. The bars are sequentially shaded on a greyscale from white -Sec, to black–Mat3. Error bars represent SDs.

Contralateral hemimandibles from mice used for micro-CT analysis were fixed in 10% formalin (Fisher Chemical SF100-4) for 48 h and then decalcified in 4.13% sodium EDTA (Fisher Bioreagents BP120) for 1 month with gentle agitation at 4°C. Hemimandibles were rinsed in PBS, dehydrated using ascending ethanol concentrations (up to 100%) and embedded in paraffin using a tissue embedder (Leica ASP300S) with a 12-h cycle. The paraffin blocks were carefully oriented and sectioned with a microtome (Leica E61160) in the sagittal plane at a 7 µm thickness. The sections for histological processing were selected based on the following criteria: The section must contain 1) roots of all molar teeth, 2) an intact cervical loop and 3) an intact ameloblast layer containing all stages of amelogenesis (Figure 1A-the staining figure?). These criteria ensure that the landmarks (molars) can be accurately related to the developmental stages of incisal ameloblasts in the histological sections. The presence of molar roots in the histological sections allowed for the correlation with corresponding micro-CT reconstructions to determine the correct position of each stage of amelogenesis in relationship to the molar teeth and to the enamel densification and thickness profiles. Selected sections were stained using a Trichrome Masson’s staining procedure, as previously described (Bui et al., 2022). After staining, the slides were air dried, mounted with mounting media (Thermo Scientific #4112), and observed using a light microscope (Nikon DS-Fi3) in the bright-field mode at ×4 and ×25 magnifications.

### 2.4 Micro-dissection procedure and landmarks

Mandible dissection, cleaning, and alveolar bone removal were carried out using previously described procedures (Molla et al., 2010; Houari et al., 2018). Briefly, freshly collected mouse mandibles were dissected on a cold plate under a dissecting microscope (Leica S8 APO) at ×2.5 magnification. A #11 blade was inserted at the mesial lingual side, between the bonny alveolar crest and the incisor tip. Keeping the scalpel parallel to the tooth’s longitudinal axis, the bone covering the lingual surface of the incisor was carefully removed, and special effort was made to not disturb the enamel organ of the incisor. Importantly, for successful identification of the dissection planes, the following steps were carried out: 1) Gingiva and periosteum covering the molar roots were thoroughly removed to facilitate the visualization of these landmarks underneath the alveolar bone, 2) the bone was removed only from the lingual face of the incisor and all 3 M carefully kept intact at their original positions, 3) the lingual surface was scored using a scalpel with a #11 blade in the planes transecting the chosen molar landmarks (Figure 1B) and normal to the incisor long axis (Figure 1C), 4) the incisor was removed completely from the bone socket and a 0.3 mm piece of the apical end was cut off to eliminate contamination by pulpal mesenchymal cells and the population of undifferentiated apical papilla stem cells (SCAP) at the cervical loop (Figure 1B). Finally, strips of enamel organ containing ameloblasts were collected from five different segments, including: 1) Secretory stage (*sec*), 2) mixture of late secretory, transition and very early maturation stage (*trans*), 3) early maturation stage (*mat1*), 4) mid maturation stage (*mat2*) and 5) late maturation stage and protective ameloblasts (*mat3*) using the identified landmarks. Between collections, mandibles were rinsed twice with PBS (Gibco) to eliminate cross-contamination between fractions and to maintain cell osmolarity. Strips of the same kind were pooled from six animals for qPCR analysis.

### 2.5 RT-qPCR analysis

Pooled strips of enamel epithelium, acquired as described in the preceding section, were immersed in ice-cold TRIzol^®^ Reagent lysis buffer (Ambion, Life technologies) and homogenized in a Motorized Tissue Grinder using RNAse-free pestle (Fisher). The RNA fraction was isolated with a chloroform and isoamyl alcohol (Sigma C0549) solution (24:1), mixed with 70% ethanol and then purified with a PureLink RNA Mini Kit (Invitrogen), following manufacturer’s instructions. Roughly two-hundred ng of total RNA was obtained from ten to twelve hemimandibles. The RNA quantities were determined using a NanoDrop One C (Thermo Scientific) spectrophotometer. The collected samples were subjected to reverse transcriptase reaction using SuperScript IV enzyme (Thermo Fisher) for 20 min at 50°C. Real-time PCR analyses were performed with StepOne Plus Real Time PCR system (Applied Biosystems) and PowerTrack™ SYBR Green Master Mix (Thermo Fisher). Cycles comprised 30 s at 95°C and 60 s at 60°C and standard melting process at the end of 40 cycles. PCR was performed on the five pooled samples in quadruplicates and the levels of expression of three major genes coding for enamel matrix proteins (EMPs) were assessed using the following primers:

*Amelx* (forward primer F: AAG​CAT​CCC​TGA​GCT​TCA​GA, reverse primer R: ACT​GGC​ATC​ATT​GGT​TGC​TG, amplicon size 371 bp),

*Enameli*n (F: TCC​TTG​TTT​TCC​TGG​GTC​TG, R: ATC​CAT​TGG​GTA​CTG​GTG​GA, 246 bp)

*Odam* (F: TTG​ACA​GCT​TTG​TAG​GCA​CA, R: GAC​CTT​CTG​TTC​TGG​AGC​AA, 197 bp).

Expression levels of the EMP genes were compared with reference gene β-actin (F: GGG​AAA​TCG​TGC​GTG​ACA​TC, R: GCGGCAGTGGCCATCTC, 76 bp).

### 2.6 Data presentation and statistical analyses

**FIGURE 2:**
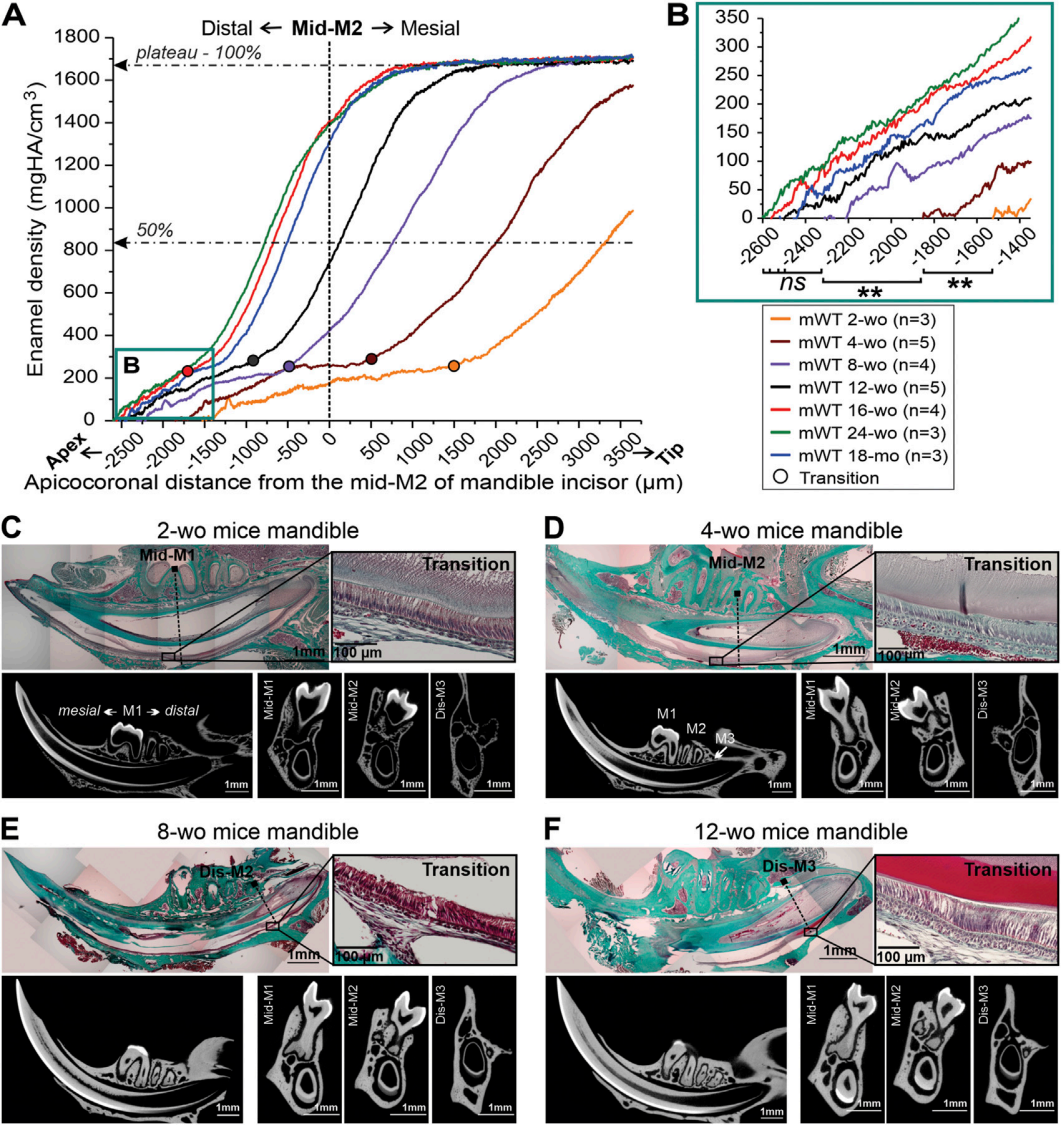
Distal movement of the transition stage and the onset of enamel mineralization in mandibular incisors of male mice of differenct ages. **(A)** Mean enamel MD profiles in incisors from mice of different ages, aligned with mid-M2 at point 0. The dotted lines intersect 50% and 100% maximum mineral density. **(B)** A close-up of the onset of incisal enamel mineralization in mice of different ages, selected in box B in Figure 2A; **: *p*-value <0.01, ns, not significant. **(C–F)** Trichrome Masson stained sections of hemimandibles, showing the relationships between transition stage ameloblasts, identified based on the morphology and molar positions in mice of different ages and the corresponding micro-CT images in midsagittal and three transverse planes intersecting the molar landmarks. **(C)** 2-week-old (2-wo), **(D)** 4-week-old (4-wo), **(E)** 8-week-old (8-wo) and **(F)** 12-week-old (12-wo); M1, M2, M3: first, second and third molar, mes-, mid-, dis-; mesial, middle, distal; wo, week-old; mo, month-old.

Enamel mineral density was expressed as mean values (sample numbers for each age group are indicated in Figure 2A). Distances and relationships between anatomical landmarks were calculated using Fiji package (ImageJ). Gene expression levels (mean ± SD) were obtained from four independent samples for each segment of amelogenesis. Statistical analyses were carried out using ANOVA with post-hoc testing for comparisons of the onset of amelogenesis and density profiles between different age groups (GraphPad nine software: Version 9.4.1). OriginLab software (2017) and CorelDraw (2020) were used to generate graphs and figures.

## 3 Results

### 3.1 Identifying stages of amelogenesis in 12-week-old mice using a combination of skeletal landmarks (positions of molar teeth) and the histological appearance of ameloblasts

We chose 12-wo male mice for our initial determination of skeletal landmarks of different stages of amelogenesis, since at this age mice approach skeletal maturity (Farooq et al., 2017; Wei et al., 2017). Identification of secretory, transition and maturation-stage ameloblasts in 12-wo mice in histological sections was based on the morphological characteristics of each stage, as outlined here. Specifically, secretory stage is the most proximal to the cervical loop of the incisor. Secretory ameloblasts are tall columnar cells and feature a specialized secretory apparatus–Tomes’ process (TP) at the distal pole facing enamel (Figure 1A). During the secretory stage, the enamel thickness increases, while it remains constant during the maturation stage. Maturation stage ameloblasts are shorter and lack TPs. The proximal poles of secretory ameloblasts interface with stratum intermedium (SI), a thin layer of flattened or cuboidal cells, which in maturation develops into a thick papillary cell layer containing a dense capillary network (CP) (Figure 1A) (Garant and Gillespie, 1969). The organic content of secretory enamel is high and it becomes significantly lower at the maturation stage, leading to weaker histological staining (Figure 1A). The transition stage is the intermediate stage between secretion and maturation. Transitory ameloblasts are shorter than secretory ameloblasts and, although they lose their Tomes’ processes, they still secrete the outer enamel layer. The transition stage length is roughly 70 µm consisting of 22.6 ± 2.8 cells (Bui et al., 2022) (Figure 1A).

Micro-CT analysis was conducted on five hemimandibles from 12-wo male mice. Using the micro-CT data, we calculated changes in enamel volume (EV) and mineral density (MD) during amelogenesis (Figure 1B; Supplementary Figure S1). The incisal EV increased sharply from the cervical loop toward the mesial root plane of the third molar (M3) for the first 2 mm, before leveling off (Figure 1B; Supplementary Figure S1A). The mineral density in all samples followed a sigmoidal pattern, with the initial apical segment of low MD, corresponding to the phase of a steep increase in EV, followed by a segment with a high densification rate, starting at ∼2 mm from the cervical loop and with a plateau at ∼1,700 mg HA/cm^3^ at roughly 3.5–4 mm from the cervical loop (Figure 1B; Supplementary Figure S1B). No differences were observed in EV and MD profiles between animals on a hard diet (mWT-4H and mWT-5H, see Supplementary Figure S1) and the rest of specimens, fed a mixed diet of hard pellets and gel food (mWT-H/G). Further micro-CT analysis revealed that positions of the molar teeth are highly consistent among specimens. Specifically, the distance from the onset of dentin mineralization to the distal plane of M3 is 1,646 μm, SD = ± 19 μm, to mid-M2 2,498 μm, SD = ± 24 µm and to mid-M1 3,434 μm, SD = ± 64 μm, (Figures 1B, C).

Based on our histological findings and micro-CT analysis, we have used molar teeth as landmarks to separate enamel epithelium into five segments, corresponding to different stages of amelogenesis (Figures 1A–C). The first (*sec*) segment is characterized by a steep increase in EV and low MD (Figure 1B). During secretory stage amelogenesis, the full thickness of enamel is deposited, but only a small fraction of mineral is present at this stage, which is why this segment was assigned to the secretory stage of amelogenesis (*sec*). The secretory nature of ameloblasts in this segment was also confirmed with histology (Figure 1A). This *sec* segment containing pure secretory ameloblasts was selected for strip dissection starting at 0.3 mm from the onset of dentin mineralization (DM) and ending 0.3 mm distal of the distal plane of M3 (Figures 1A–C). Using histological sections, we were able to determine the onset of the transition zone at ∼0.17 mm distal to M3 (or ∼1 mm distal to Mid-M2) (Figure 1D; Table 1) and is ∼0.07 mm long. To avoid possible contamination with transition stage ameloblast, the last 0.13 mm of secretory stage cell layer were collected in the next strip. This strip, called *trans*, extended from 0.3 mm distal to M3 to mid-M2 and included the late secretory, whole transition and very early maturation stages (Figures 1A–C). At the distal boundary of this zone, the densification rate is low and similar to zone 1 (*sec*), containing early and mid-secretory ameloblasts. However, roughly 2 mm from the onset of DM, the densification rapidly intensifies to its highest rate of ∼810 ± 92 mg HA/cm^3^ per mm (Figure 1B). The increase in the rate of densification corresponds to the plateauing of the enamel volume (Figure 1B). This zone exhibits a large variation among samples in both EV and MD (Figure 1B; Supplementary Figure S1), which is likely due to the fact that the exact position of transition zone varies between the specimens (Table 1). The next strip contained the early maturation stage (*mat1*) and extended from mid-M2 to mid-M1 (Figures 1A–C). The rapid rate of densification is similar to second half of *trans*. At this stage, all samples have reached full enamel thickness and the densification rate is high. The mid-maturation segment (*mat2*) extended 1.5 mm mesial to mid-M1. Cells in this fraction are characterized by a strong yellowish-brownish hue, due to the intense iron transport during this stage (Reith, 1961; Wen and Paine, 2013). The late maturation/protective ameloblast layer (*mat3*) was collected from the end of *mat2* to the point of eruption. The cells in this layer are transparent, suggesting that iron transport is associated with *mat2* only. In summary, the landmarks associated with different stages of amelogenesis, based on the positions of molar teeth, can be easily determined (Figure 1D). Importantly, these landmarks allow for the microdissection of the incisal enamel organ into five strips of similar length (∼1.5 mm), which provided sufficient amounts of material for gene and protein expression studies, when strips from five to six mice were pooled together.

**TABLE 1:** Distances from the onset of dentin mineralization (Apex) to important milestones of amelogenesis in mandibular incisors of mice of different ages, outlined in Figure 3C. The last row represents maximum rates of enamel densification in mandibular incisors of mice of different ages, plotted in Figure 3D.

Distance (µm)	Age						
	2-wo	4-wo	8-wo	12-wo	16-wo	24-wo	18-month
Apex to mid-M2	1,722 ± 100^a^	2,056 ± 97	2,450 ± 190	2,494 ± 122	2,588 ± 164	2,676 ± 208	2,442 ± 305
Apex to transition	3,234 ± 97	2,486 ± 193	1,944 ± 296	1,476 ± 293	0950 ± 251	0916 ± 78	1,042 ± 231
Apex to 50% mineralization	4,918 ± 019	4,066 ± 114	3,281 ± 289	2,492 ± 329	1,965 ± 329	1,982 ± 446	2,046 ± 254
Apex to 100% mineralization	6,816 ± 252	7,135 ± 083	5,011 ± 328	4,544 ± 270	3,647 ± 737	4,558 ± 930	3,648 ± 537
Maximum rate of densification (mg HA/cm^3^ per mm)	453 ± 68	550 ± 46	653 ± 80	811 ± 92	1,023 ± 132	1,010 ± 212	981 ± 122

^a^ Standard deviation.

### 3.2 Analysis of gene expression levels of micro-dissected incisal epithelium from 12-wo male mice

Figure 1D shows a 12-wo mouse mandible during the micro-dissection process. To guide this process, markings were placed at relatively equal intervals of ∼1.5 mm along the ameloblast layer (Figure 1D, inset). The strips of the same kind from 12 mandibular incisors of six individual mice were pooled together to obtain at least 20 ng/μL total RNA per stage. *Mat2* and *mat3* equally contained the least RNA quantity, about 2.1 and 1.6 times less, respectively, compared to *sec* and *trans* fractions (data not shown).

To assess the accuracy of our landmarking, we have conducted analyses of EMP gene expression in different segments of the incisal enamel organ, collected as described above. Ameloblasts express different sets of genes, based on the stage of amelogenesis (Lacruz et al., 2017), which can be used as markers of specific stages of amelogenesis. For our studies we have chosen two secretory stage genes–*Enam* and *Amelx*, and one maturation stage gene - *Odam*. Expression of the three EMP coding genes was compared to the expression of reference gene *β-actin* and the expression data was normalized by calculating ratio of the gene of interest to *β-actin* and presented as the mean ratio ± SD (Figure 1E). The *Amelx* expression level was highest in *sec* samples, 553.6 ± 143 times higher than *β*-actin expression and was 85-times higher than that in the maturation segments (*mat1-3*) (6.5 ± 5.5, *Amelx*/*β-actin* ratio) and almost 5 times higher than in the *trans* segment (Figure 1E; Supplementary Table S2). *Enam* expression levels were about 5% of the *Amelx* expression level, which is consistent with the fact that Amelx constitutes 90% of secretory enamel matrix. Similarly to *Amelx*, *Enam* levels were 100-times higher in the *sec* strip than in the *mat* segments (26.8 ± 2.2 vs. 0.27 ± 0.17 *Enam*/*β-actin* ratio) and 6.4-times higher than in the *trans* strip (Figure 1E; Supplementary Table S2). In contrast, *Odam* expression was higher in the maturation stage, with no significant differential expression between the three portions of *mat1*, *mat2*, and *mat3*, with an average level of 121.8 ± 22.5 *Odam*/*β-actin* ratio. The *sec* segment expressed *Odam* at 11.5 times lower level than *mat1-3*, with a 10.5 ± 4.8 *Odam/β-actin* ratio (Figure 1E; Supplementary Table S2)*.* Likewise, *Odam* levels in the *trans* segment that contains late secretory, transition and very early maturation ameloblasts was 5.6 times higher than in the *sec* strip. Overall, these results indicate that our landmarking method allows one to obtain with high accuracy segments of the incisal enamel organ, corresponding to different stages of amelogenesis.

### 3.3 Distal movement of incisal apices and transition stage of amelogenesis in mandibles in spatial relationship to molars over the lifespan of mice

**FIGURE 3:**
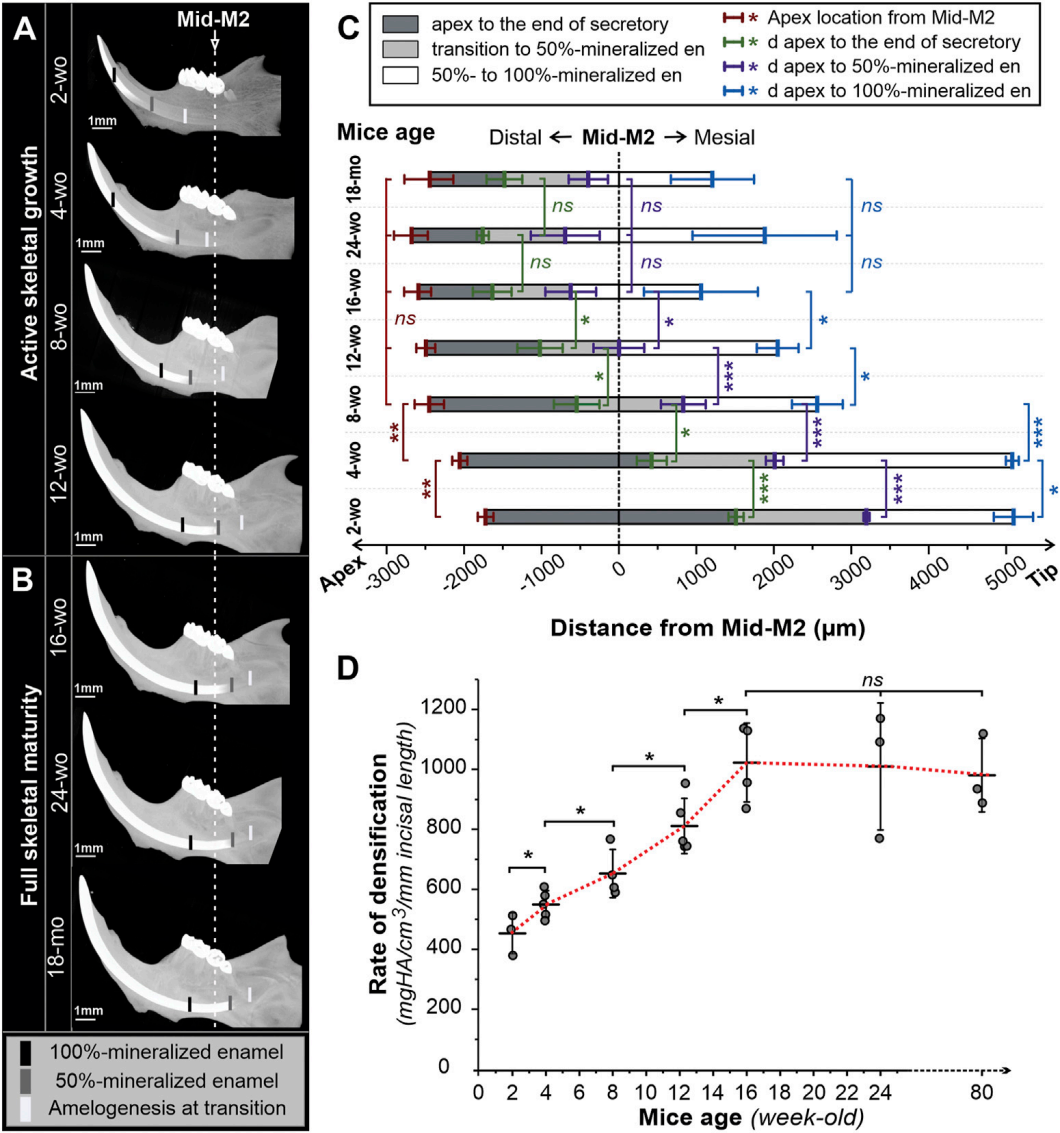
3D reconstructions of micro-CT scanned mandibles of male mice of different ages. Lingual view images of hemimandibles of mice of different ages during active skeletal growth **(A)** and at skeletal maturity **(B)**. Black, grey, and white marks indicate, respectively, the positions of fully mineralized (100%), half-mineralized (50%) enamel, and transition secretory-transition boundaries aligned to the second molar (Mid-M2) plane. **(C)** Sequences of amelogenesis in mandibular incisors in mice of different ages aligned in the mid-M2 plane. Mean distances (in µm) from the apex to the position of secretory-transition boundary (STB) (dark grey), partially mineralized enamel matrix (50% mineral density) (grey), and fully mineralized enamel (100%) (white) were presented; the positions were aligned with mid-M2. Error bars represent ± SD values; red -apex positions, green-STB, purple—50% mineralized, blue—100% mineralized **(D)** Maximum rate of enamel densification, expressed as mean ± SD (mineral density per mm incisal length). wo, week-old; mo, month-old; *, **, and *** reflect significance levels with *p*-values <0.05, 0.01, and 0.001, respectively, ns, not significantly different.

It is a well-known fact that during craniofacial growth the apices of murine incisors undergo distal movement, however, the exact dynamics of this process are not well understood. We have conducted a systematic study of this process using a combination of histology and micro-CT. Seven groups of WT mice of different ages, from 2-wo to 18-mo, were studied. Enamel density and ameloblast morphology in mandibular incisors were analyzed to determine spatial relationships between different stages of amelogenesis with respect to molar teeth (Figure 2). Averages of mineral density of at least three animals per each age group, were aligned using the mid-M2 plane as a reference point (Figure 2A). We found this to be a valuable reference point that allows for the alignment of density profiles of different age groups, regardless of differences in incisor length and curvature. Based on the analyses of histological sections, as described in the preceding section, we have identified the onset of the transition stage for all age groups (Figures 2C–F) and marked them on the plot in Figure 2A. These studies revealed a clear trend of distal movement of the transition zone with age. In younger animals, the transition zone was located in the anterior region: 1,512 ± 97 µm mesial to mid-M2 in 2-wo mice and 424 ± 193 µm mesial to mid-M2 in 4-wo mice (Figures 2A, C, D, 3A–C; Table 1). In 8-wo mice, the position of transition was 546 ± 295 µm distal to mid-M2 (Figures 2A, E; Table 1). The position of the transition zone in 12-wo mice is located 1,020 ± 293 µm distal to mid-M2 (Figures 2A, F; Table 1) and in 16-wo and older animals it is located at 1,626 ± 218 µm distal to mid-M2 (Figure 2A; Table 1). These data indicate that the transition zone moves more than 3 mm distally from the time of eruption of first and second molars and incisors in 2-wo mice to mice at full skeletal maturity.

Moreover, not only does the transition zone move apically with age, but the apex of the incisor and the onset of enamel formation also recede distally from the molars (Figures 2B, 3C; Table 1). The onset of enamel mineralization in 2-wo is 1,722 ± 100 µm away from the mid-M2, while it moves significantly (*p* < 0.01) further away to 2,056 ± 97 µm in 4-wo and 2,450 ± 190 µm in 8-wo mice, respectively (Figures 2B, 3A–C; Table 1). As shown, for mice older than 8-wo, this distal movement of the apices is still occurring, but these changes are not significant. However, in older mice, higher variation within each group was observed: *e.g.*, 2,588 ± 164 µm in 16-wo group (*n* = 4) versus 2,676 ± 208 and 2,442 ± 305 µm in the 24-wo (*n* = 3) and 18-mo (*n* = 3) groups, respectively.

### 3.4 Differences in the rate of mineralization in incisal enamel in mice of different age

In all age groups, densification of incisal enamel followed similar sigmoidal patterns, as was shown for 12-wo animals (Figure 1B; Supplementary Figure S1B), with slower densification rates in the apical portions, corresponding to the secretory stage of amelogenesis, followed by rapid densification in transition and early maturation stages, and reaching a plateau at late maturation stage (Figure 2A). Of note, although the overall shapes of the curves were similar, there were significant differences in terms of the lengths of these different stages. More specifically, the length of the secretory stage, defined as a distance from the onset of enamel mineralization to transition zone was longer in younger mice than in the older animals (Figure 2A; Table 1).

To assess the differences in dynamics of amelogenesis in mice of different ages, we have studied densification of enamel in micro-CT 3-D reconstructions of the mouse mandibles. Figure 3A comprises micro-CT images of mandibles of actively growing mice two- to 12-wo, while Figure 3B presents mandibles of older skeletally mature mice, ages 16-wo to 18-mo. All mandibles are presented in the same orientation, at the same magnification, and are aligned relative to the mid-M2 transverse plane. Average positions (n ≥ 3) of the transition stage of amelogenesis at half (50%) and maximum (100%) enamel density are marked in the images in Figures 3A, B. Distances from the apex to the transition zone at half- and maximum density positions with respect to the mid-M2 plane were measured and plotted in Figure 3C and shown in Table 1.

Visual comparison of the micro-CT images clearly shows the distal movement of the cervical loops with respect to mid-M2 and elongation of the incisors with age (Figures 3A, B), confirming the results discussed in the preceding paragraphs. In addition to a longer secretory phase, the rate of densification in younger animals is much slower than in older animals (Figures 2A, 3A, B, D; Table 1). Hence, maturation stage ameloblasts that complete mineralization do so at a slower rate in young mice compared to that in older mice. This is readily visible when 2-wo and 4-wo mice are compared to 8-wo and older mice (Figure 3A). However, it was the 4-wo group and not the youngest 2-wo group that had the longest mineralization process (7,135 ± 83 μm and 4,066 ± 114 μm, respectively, from the apex to 100% and 50% MD) in the incisor (Figure 3C; Table 1). This might be explained by the fact that, as in 2-wo mice, the maximum enamel MD is only 1,574 ± 35 mgHA/cm^3^ (data not shown), which is ∼94% of maximum MD observed in mice of 4-wo and older mice, when the enamel layer reached a plateau of densification at a greater density of ∼1,670 mgHA/cm^3^ (Figure 2A). The maximum densification rate, represented by the linear segment of the densification curve, associated with early maturation stage (Figures 1B, 2A), increased significantly with age and reached maximum in 16-wo mice, resulting in significant shortening of incisor mineralization (Figures 2A, 3; Table 1). From this age on, the dynamics of incisal growth stabilized, as indicated by the lack of differences in positions of all three marks, and the maximum rate of densification between 16-wo, 24-wo, and 18-mo groups (Figures 3B–D; Table 1). Importantly, mice reach skeletal maturity at 14 weeks of age, while the changes in the craniofacial region occur in the first 4 weeks after birth (Wei et al., 2017). Our observations suggest that the changes in the positions of the incisors in the mandibles and the dynamics of amelogenesis correlate with postnatal craniofacial growth. Of note, standard deviations among individuals within the same group were smaller in younger mice, *e.g*., 100% mineralization occurred at 7,135 ± 83 µm from the apex in 4-wo, while this level was reached at 5,011 ± 328 μm and 3,647 ± 737 µm in 8-wo and 16-wo, respectively (Figure 3C; Table 1). These findings suggest that the noted landmarks are more reliable in younger mice.

As a consequence of the distal movement of the apices and the transition zone, as well as changes in the rate of mineralization, when micro-dissecting mandibular incisors in different age groups, different landmarks need to be used. Specifically, in 2-wo mice, the secretory-transition boundary (STB) is located in the plane transecting the mid-M1 plane (Figure 2C). The STB is in the plane of mesial M2 root in 4-wo animals (Figure 2D). In 8-wo mice, STB moves further distally, and it starts in the distal plane of M2 (Figure 2E). As forementioned, STB micro-dissection landmarks shifted apically by ∼0.3 mm from distal M3 in 12-wo. In 16-wo, the STB position varies between 0.44 and 0.97 mm distally to the distal M3 plane or 1,637 ± 251 µm from mid-M2, as shown in Figure 3C. The position of STB in older mice (24-w and 18-month) is similar to that in 16-wo mice (Figures 2A, 3B, C; Table 1), suggesting that the mice reach full skeletal maturity by 16 weeks.

## 4 Discussion

It is common knowledge that positions of murine mandibular incisors and their spatial relationships with molar teeth change with age. As reported here, we have found that throughout the active skeletal growth phase (weeks 2–12) the apices of the incisors and the onset of enamel mineralization move distally in relation to molar teeth. The position of the transition stage also moves distally. We also found in 16-wo and older mice that incisor length and their positions relative to molar landmarks remain unchanged, indicating that the magnitude of changes in incisors correlates with dynamic skeletal growth. The present study emphasizes the need to address the importance of age in experimental design, when studying amelogenesis and the etiology of enamel defects using mouse incisor models and when comparing results from different laboratories. The magnitude of changes in mandibular incisors is greatest at the younger ages, when overall skeletal growth rate is the highest (Farooq et al., 2017; Wei et al., 2017) in comparison to older animals. These changes include: 1) Changes in length and position of developmental stages of amelogenesis with age, 2) distal drift of incisal apices during the active skeletal growth phase, 3) the amelogenesis process shortens with age, which is quite an interesting observation, since incisors in 2-wo mice are roughly 1 mm shorter than in skeletally mature mice, and 4) changes in rates of enamel volume acquisition and densification. Based on these facts, we believe that 12-wo mice could be considered an optimal model for fresh micro-dissection and ameloblast collection when the goal is to compare different stages of amelogenesis. The 12-wo incisor provides an ameloblast layer of sufficient length, which lends itself to easier handling and manageable micro-dissection. Moreover, at this age, the landmarks corresponding to different stages of amelogenesis are easily identifiable, which allows for better reproducibility of dissections (Figures 1, 2A, F).

To assess the accuracy and reliability of our landmarking methodology, we conducted expression analyses of genes encoding key EMPs, with distinct expression profiles in five micro-dissected segments of incisal enamel organs of 12-wo mice. The observed expression profiles are consistent with our current understanding of EMP expression in developing mouse enamel. Amelogenin (*Amelx*) the major protein in enamel matrix (>90%), is highly expressed in presecretory and secretory stages of amelogenesis (Bleicher et al., 1999; Lacruz et al., 2017). Similarly, enamelin (*Enam*) is highly expressed in the secretory stage (Gallon et al., 2013), while odontogenic ameloblast-associated protein (*Odam*), also known as Apin, is expressed by late secretory and maturation stage ameloblasts (Park et al., 2007; Moffatt et al., 2008). The expression profiles obtained in the current study are consistent with these literature reports, which demonstrate the practicality and high fidelity of our landmarking methodology. Several landmarks to assist in identification of the stages of amelogenesis in continuously growing murine incisors have been suggested for mice (Molla et al., 2010; Houari et al., 2018) and for rats (Smith and Nanci, 1989; Smith et al., 1996; Moffatt et al., 2008; Lacruz et al., 2012a). However, this is the first time that a micro-dissection approach to study mouse amelogenesis in mice of different aged was designed to achieve 1) high accuracy and confirmation by multiple approaches (i.e., micro-CT, cell morphology and gene expression profiling) and 2) maximization of collected sample yield, as the whole ameloblast cell layer is collected, which can help reduce sample size, while making multiple types of analyses possible. This approach provides means to maximize information, such as the assessment of the behavior of ameloblasts during the intermediate stage between secretory and maturation stages, which is excluded in the previously reported methods (Lacruz et al., 2010; Jedeon et al., 2014). It also allows one to compare the behavior of maturation stage ameloblasts in different parts of forming enamel, for example, between the transition stage and the very beginning of maturation, to *mat3*, which consists of protection stage ameloblasts, and to correlate these stages of histodifferentiation with profiles of EV and MD, obtained using a high-resolution micro-CT. It is important to emphasize that although this study provides an accurate guide to the positions of different stages of amelogenesis in mouse incisors with respect to molar teeth, future more in-depth studies, which similarly use a combination of histology, expression analysis and micro-CT, may be needed for mice of ages other than 12-wo if more precise information is needed for a given age group. It should also be noted that this study has been conducted on WT mice and that the key mechanistic dynamics of amelogenesis and the effects of aging illustrated here are likely to be different from those seen in genetically modified mouse models that result in altered enamel formation.

## Data Availability

The original contributions presented in the study are included in the article/Supplementary Material, further inquiries can be directed to the corresponding author.
