# Supplementary material for: Identification of stages of amelogenesis in the continuously growing mandiblular incisor of C57BL/6J male mice throughout life using molar teeth as landmarks
Source: Front Physiol. 2023 Feb 10;14:1144712. doi: 10.3389/fphys.2023.1144712 (PMC9950101; doi:10.3389/fphys.2023.1144712)
Supplement: Supplementary file 1 [file DataSheet1.PDF]

Supplementary data:

Figure S1:

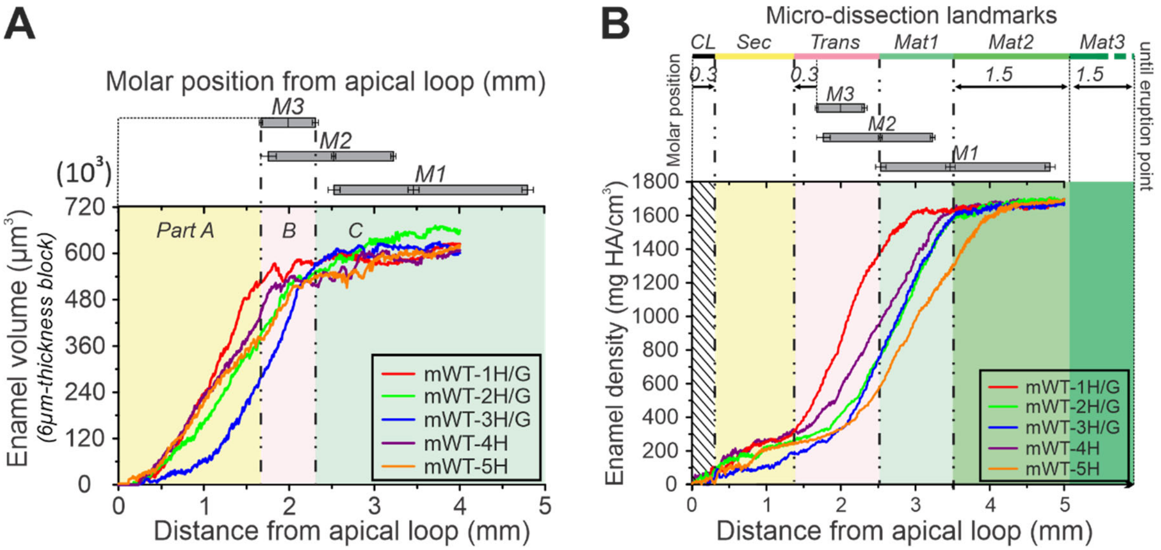

Figure S1: Mineral profile in mandibular incisors of twelve-week-old mice. Enamel incisors from 12-wo mice, raised with different diets, hard chows (H) or a combination of hard chows and soft gel food (H/G), were scanned with high resolution micro-CT (6 µm pixel) and analyzed to assess (**A**) the enamel volume (EV) profile (in μm^3) and (**B**) the mineral density (MD) profile (in mg HA/cm^3) from the apex toward the tip. Molar positions, including M1, M2 and M3, were expressed as the Mean ± SD µm from the apex. The scan plane was perpendicular to the incisor long axis at mid-M2 and the zone of observation (0 – ~4 to 5 mm) was limited to minimize the impact of incisor curvature on the EV calculation; *sec*: pure secretory, *trans*: mixture of small portion of late secretory, the whole transition stage and mostly very early maturation, *mat1*: early maturation, *mat2*: middle maturation, *mat3*: late maturation/protection, mWT: male wild-type mice, CL: cervical loop.

Table S1: List of mice used.

Mice source	mWT C57BL/6J<br>in-bred at U. of Pittsburgh	mWT C57BL/6J<br>ordered from Jackson lab
Fed with	Mixed hard chow and gel food	Only hard chow
**Mice used for micro-CT and histology staining (n=27)**		
Step 1: Compare enamel densification profile of in-bred mice and commercial mice.		
12-wo	3	2
16-wo	2	2
Step 2: Analyze enamel densification profile and amelogenesis of in-bred mice.		
2-wo	3	0
4-wo	5	0
8-wo	4	0
24-wo	3	0
18-mo	3	0
**Mice used for micro-dissection and RNA collection (n=24)**		
Step 3: Select a mice age to perform experiment for checking chosen landmarks.		
12-wo	24	
**Total of mice used: n=51**		
mWT: male wild-type; n: number of mice used; wo: week-old; mo: month-old.		

Table S2: Gene expression level of Amelx, Enam and Odam in incisal dental epithelium of 12-week-old male wild-type mice.

Gene	*β-actin*				*Amelx*				*Enam*				*Odam*			
**mRNA expression level – Raw data (Cycles)**																
**Replicons**	**1**	**2**	**3**	**4**	**1**	**2**	**3**	**4**	**1**	**2**	**3**	**4**	**1**	**2**	**3**	**4**
Sec	21.78	21.52	21.70	21.84	12.09	11.10	10.78	12.82	16.93	16.83	16.90	16.92	18.46	17.92	17.87	19.06
Trans	21.91	21.34	21.40	21.97	15.41	14.12	14.03	14.78	19.98	20.06	20.19	18.96	15.45	15.80	15.76	15.76
Mat1	21.89	21.71	21.94	21.75	19.61	17.67	17.56	21.33	23.98	23.10	23.41	24.94	14.90	14.73	14.90	14.92
Mat2	21.91	21.69	22.06	22.21	20.75	19.45	17.69	18.59	23.58	23.25	24.47	25.02	15.01	14.99	15.19	15.22
Mat3	22.03	21.73	21.96	22.38	19.63	16.41	19.56	20.62	24.92	23.29	25.30	24.51	15.45	14.95	15.15	15.46
**mRNA expression level - Relative ratio compared to β-actin (times)**																
**N=4**					**Mean**		**SD**		**Mean**		**SD**		**Mean**		**SD**	
Sec					553.61		143.00		26.81		2.21		10.56		4.89	
Trans					115.02		45.00		4.22		2.68		59.52		3.90	
Mat1					7.88		7.60		0.28		0.13		130.30		33.95	
Mat2					5.99		3.19		0.24		0.09		118.10		24.20	
Mat3					7.58		6.20		0.30		0.29		116.96		9.49	
**Change of gene expression during amelogenesis (secretory set as 100%)**																
Sec					100.00				100.00				100.00			
Trans					20.78				15.74				563.64			
Mat1					1.42				1.04				1233.90			
Mat2					1.08				0.90				1118.37			
Mat3					1.37				1.12				1107.58			
